# The impact of chronic exposure to cigarette and waterpipe smoke extracts on telomeres, telomerase, and cancer progression

**DOI:** 10.3389/fgene.2026.1837683

**Published:** 2026-09-09

**Authors:** Daria Azar, Danya Kabbani, Carole Abdel Karim, Reem Akika, Pamela Melki, Mario El Hourani, Rola Salman, Farah J. Nassar, Alan Shihadeh, Akram Ghantous, Rihab Nasr, Nathalie Khoueiry-Zgheib

**Affiliations:** 1 Department of Pharmacology and Toxicology, Faculty of Medicine, American University of Beirut, Beirut, Lebanon; 2 Alice Ramez Chagoury School of Nursing, Lebanese American University, Byblos, Lebanon; 3 Department of Mechanical Engineering, Maroun Semaan Faculty of Engineering and Architecture, American University of Beirut, Beirut, Lebanon; 4 Environment and Lifestyle Epidemiology Branch, International Agency for Research on Cancer, Lyon, France; 5 Department of Anatomy, Cell Biology, and Physiological Sciences, Faculty of Medicine, American University of Beirut, Beirut, Lebanon

**Keywords:** cancer cell lines, cigarette smoking, waterpipe smoking, chronic exposure, telomeres, telomerase, EMT, migration

## Abstract

**Introduction:**

Tobacco smoking remains a leading cause of many life-threatening diseases, particularly lung cancer. Waterpipe smoking has rapidly gained popularity worldwide, while research on this tobacco form remains limited. Tobacco smoking has been linked to the shortening of telomeres and activation of telomerase, important regulators of genomic stability and cancer progression. However, the direct impact of whole-smoke exposure on telomere dynamics and its relationship with cancer progression remains minimally explored, and this requires suitable experimental systems.

**Methods:**

This study aims to demonstrate an experimental procedure to enable prolonged exposure of lung cancer cells (A549) to sub-toxic concentrations of cigarette and waterpipe smoke extracts, and to investigate in those cells the effects on telomere length, telomerase activity, and cancer progression. We also checked telomere length, epithelial-to-mesenchymal transition (EMT) markers, and migration in two other colon (HCT116) and breast cancer (MCF7) cell lines.

**Results:**

After 12 weeks of exposure, there was a significant decrease in relative telomere length in heterogeneous/parents of A549 cells with waterpipe exposure, and a significant increase in telomerase activity with cigarette exposure. Chronic exposure to both smoke extracts also led to a significant upregulation in the expression of the SNAI2 mesenchymal marker in both heterogeneous populations and single-cell-derived colonies. Additionally, chronic exposure to cigarette smoke extract significantly enhanced migration in single-cell-derived colonies. Comparable results were seen in the colonies of colon and breast cancer cell lines.

**Discussion:**

Our findings revealed that chronic smoke exposure disrupts telomere dynamics particularly in lung cancer cells and triggers transcriptional EMT changes that might contribute to cancer progression in lung, breast, and colon cancer cells, offering new insights into a potential link between tobacco smoke, telomere dysfunction, and cancer progression.

## Introduction

Tobacco smoking is a common practice, and it imposes a major global health burden. The World Health Organization (WHO) estimates that 1.3 billion people worldwide smoke tobacco products, and that each year more than seven million people die because of it (Smoking, Our World in Data, 2023). The major causes of mortality among smokers are cardiovascular diseases, respiratory diseases, cancer, and diabetes (Lariscy et al., 2018). Lung cancer is one of the most common and deadliest forms of cancer worldwide, responsible for 1.8 million deaths worldwide in 2020. 85% of lung cancer cases are primarily caused by tobacco smoking (Lung Cancer, 2026).

Waterpipe smoking has become a global epidemic in the past two decades, with the highest prevalence in Middle Eastern (10.7%) and European countries (10.9%). It has also become especially common among adolescents and young adults (10–24 years) in the United States, Middle East and the European region (Sepidarkish et al., 2025). With the lack of country-specific policies and regulations on waterpipe usage (Alaouie et al., 2022), this global smoking trend raises a major health concern since waterpipe smoking has been significantly associated with chronic respiratory diseases, cardiovascular diseases, diabetes, infertility, and many types of cancers including lung, gastric, esophageal and bladder cancers (Sepidarkish et al., 2025).

Tobacco smoke contains thousands of chemicals and toxicants that enter the lungs upon inhalation and contribute to carcinogenicity, inflammation, and oxidative stress (Smoking, Our World in Data, 2023). Both waterpipe and cigarette smoke contain nicotine, tar, carbon monoxide (CO), polycyclic aromatic hydrocarbons (PAHs) which are carcinogenic compounds, and volatile organic compounds (VOCs) which are harmful for the respiratory system (Badran and Laher, 2020; Primack et al., 2016). The main difference between waterpipe and cigarette smoking lies within the duration of exposure, the smoke volume delivered, and the burning of charcoal associated with waterpipe smoking (Badran and Laher, 2020). While cigarettes may contain higher levels of some VOCs (Daher et al., 1994), waterpipe smoke contains various types of heavy metals including lead and chromium (Badran and Laher, 2020), higher levels of CO (Badran and Laher, 2020; Primack et al., 2016), and significantly higher amounts of PAHs, mostly coming from the charcoal (Daher et al., 1994; Sepetdjian et al., 2010).

Human telomeres are double-stranded (TTAGGG)n tandem repeats of DNA sequences located at the chromosomes ends and stabilized by shelterin proteins. Their role is to protect the chromosome ends against DNA damage and fusion (Lu et al., 2013; Shay and Wright, 2019; Turner et al., 2019). Telomerase is the enzyme that replicates and maintains the length of telomeres (Lu et al., 2013). Telomeres are susceptible to oxidative DNA damage, making them prone to shortening in smokers (Valavanidis et al., 2013).

Human subject studies have found a strong correlation between smoking and accelerated telomere shortening in different cell types, including small airway epithelium (Walters et al., 2014), leukocytes, and lymphocytes (Astuti et al., 2017; Wulaningsih et al., 2016; Muezzinler et al., 2015). Some studies also found an association between smoking and increased telomerase expression and activity in humans (Marcon et al., 2017; Fernandes et al., 2021; Bozkus et al., 2017). While many studies have investigated smoking and telomere biology in human subjects, only a few studies have examined the direct effects of tobacco smoke extracts on telomeres and telomerase in cell models. Notably, these studies reported a significant decrease in telomere length following 2 weeks of exposure to cigarette smoke extract in human non-small cell lung carcinoma and human lung fibroblasts (Deb et al., 2024) or cigarette smoke condensate (CSC) in mouse embryonic stem cells (Huang et al., 2013). Moreover, the effect of chronic smoke exposure, and particularly waterpipe, on telomeres and telomerase in various cell models remain poorly explored.

Telomeres and telomerase are crucial factors in the initiation and progression of cancer. Critically short telomeres result in the loss of chromosomal protection, leading to genomic instability and chromosome end-to-end fusions. This instability allows pre-cancerous cells to accumulate additional mutations, promoting their transformation into malignant cells. Conversely, telomerase is reactivated in 85% to 90% of cancer cells, enabling them to maintain telomere length, which supports continuous cell proliferation and confers an immortal phenotype (Turner et al., 2019), (Yuan et al., 2020). As such, research has demonstrated a link between telomere dynamics, telomerase activity, and lung cancer progression across different stages, as well as patient prognosis. In lung cancer patients, shorter telomeres were found to be associated with shorter patient survival (Faugeras et al., 2022), increased mortality (Doherty et al., 2018), worse clinical outcomes, and a higher relapse rate (Fernandez-Marcelo et al., 2015). Additionally, increased telomerase activity in lung tumors was associated with poor patient prognosis (Faugeras et al., 2022; Fernandez-Marcelo et al., 2015). Thus, the literature points to the fact that shorter telomeres and higher telomerase activity could be associated with a more aggressive cancer phenotype. Short telomeres confer genomic instability and promote tumor progression, metastasis and chemotherapy resistance (Deng and Chang, 2007). On the other hand, higher telomerase activity favors cancer progression not only by maintaining the telomeres at a stable short length and allowing cancer cells to proliferate indefinitely (Yuan et al., 2020; Fernandez-Garcia et al., 2008), but also by playing a role in therapy resistance (Judasz et al., 2022; Lipinska et al., 2017), and by promoting epithelial-to-mesenchymal transition (EMT) and cancer stemness (Judasz et al., 2022; Liu et al., 2013; Zhang et al., 2015). Thus, it is of our interest to study these dynamics in cancer cells and to explore how they can be impacted by chronic smoke exposure.

Given the lack of studies on the effects of smoke exposure on telomeres and telomerase in cell models and given the critical role of the telomere-telomerase dynamics in cancer progression, in this study, we sought to demonstrate a procedure to provide prolonged exposure of lung cancer cells to cigarette and waterpipe smoke extracts generated using a puffing robot, evaluate changes in relative telomere length (RTL), as well as telomerase expression and activity, in human adenocarcinoma alveolar basal epithelial cells (A549) chronically exposed to a sub-toxic concentration of cigarette smoke extract (CSE) and waterpipe smoke extract (WSE), and assess the phenotypic and molecular changes related to cancer progression following chronic exposure (Azar, 2024). We further assessed RTL, EMT markers and migration in two other cancer cell lines which are HCT116 for colorectal cancer and MCF7 for breast cancer.

## Materials and methods

### Cell lines

A549 is a human epithelial adenocarcinoma cell line, HCT116 is a human colorectal carcinoma cell line, and MCF7 is a human breast adenocarcinoma cell line. These were obtained from the American Type Culture Collection (Virginia, United States). Cells were cultured in Dulbecco’s Modified Eagle Medium (DMEM) with 10% fetal bovine serum (FBS), 1% sodium pyruvate, 1% penicillin and streptomycin antibiotic mix (Sigma-Aldrich, United States), and 0.2% plasmocin prophylactic (InvivoGen, United States). The three cell lines were authenticated using the AmpFlSTR® Identifiler® PCR Amplification Kit in Macrogen Inc. (Seoul, South Korea) and tested to be *mycoplasma* free using the MycoStrip *Mycoplasma* Detection Kit (InvivoGen, United States).

### Cigarette and waterpipe smoke extracts

Cigarette smoke was generated from 3R4F Kentucky Research Cigarettes using ISO protocol 3308 (2009) (Shihadeh and Saleh, 2005), which specifies 2-s duration puffs of 35 mL volume every 60 s until the cigarette is consumed. Waterpipe smoke was generated using the “Beirut Method”, a validated smoking protocol described by *Shihadeh et al.* (Shihadeh and Saleh, 2005), which was derived from real-world human puff topography measurements taken from 52 individuals using a waterpipe in Beirut cafés. The Beirut Method specifies 171 puffs of 2.6-s duration, 530 mL volume, and 17 s inter-puff interval, the amount of tobacco loaded in the head and charcoal application schedule, among other variables. The tobacco mixture used in this study was “two-apples” flavor, manufactured by Al Fakher (Dubai, UAE). Chemical characterization of waterpipe smoke and comparison between waterpipe and cigarette smoke constituents were previously extensively described (Daher et al., 1994; Sepetdjian et al., 2008; Al et al., 2008; Monzer et al., 2008).

Total particulate matter (TPM) produced during the waterpipe and cigarette smoking sessions were trapped on glass fiber filters (Pall Type A/E). Filters were inserted into a syringe with DMEM and pressed into a conical tube with 1% Penicillin/streptomycin, 10% FBS, and 1% sodium pyruvate added to the extracted solution. The concentration of the extract was calculated based on the number of filters, the TPM mass on each filter, and the DMEM volume used to soak the filters. We extracted for waterpipe on average 982.46 mg of TPM which is equivalent to half a waterpipe session (1 session = 1 h), and for cigarette 47.19 mg of TPM which is equivalent to 5 cigarettes. Before use, the CSE or WSE were diluted using complete DMEM to reach the Inhibitory Concentrations that induce 20% or 50% cytotoxicity (IC20 and IC50), that were used for the downstream experiments. CSE and WSE extracts were stored at −20 °C.

### MTT assay

To estimate the sub-toxic concentrations, we measured the metabolic activity of the cells using the 3-(4,5-dimethylthiazol-2-yl)-2,5-diphenyl tetrazolium bromide (MTT) assay (Sigma-Aldrich, United States). We seeded the cells at a density of 6000 cells per well as triplicates in a 96 well-plate and incubated for 24 h, after which they were exposed to a wide range of concentrations of CSE (0, 0.1, 0.5, 0.7, 1.0, 1.5, 2.0 mg/mL) and WSE (0, 1, 3, 5, 7, 10, 12,15, 20, 25, 30 mg/mL). DMEM was used as control. After 24 h of exposure, MTT was added at 0.4 mg/mL final concentration to the cells and incubated for 4 h. Then, 200 µL DMSO was added to measure the optical density at 595 nm with an enzyme-linked immunosorbent assay (ELISA) plate reader. Based on the dose response curves, IC20 and IC50 were calculated.

### Trypan blue assay

Trypan blue assay was used to measure cell viability. Cells at a total number of 2-3 x10^5^ were seeded in a 6-well plate. 24 hours post exposure to WSE and CSE, the collected cells were counted using trypan blue dye (Sigma-Aldrich, United States). The percentage of cell viability, and concentration of total and alive cells/mL were determined using an automated cell counter TC20 (BioRad, Germany).

### Apoptosis assay

Apoptosis assay using the Annexin V-FITC Early Apoptosis Detection Kit (Cell Signaling Technology, United States) was done as per the manufacturer’s protocol. After staining, the samples were run on a flow cytometer, Guava EasyCyte 8, and data were analyzed using the InCyte software.

### Cell cycle analysis

Propidium iodide (PI) (Sigma-Aldrich, United States) based flow cytometry allowed the detection of cells in Sub G0 and any potential cell cycle arrest at 24 h. Control and treated cells were first collected then washed with 1X PBS. Fixation was done using 1:4 PBS and 100% ethanol and then cells were stored at −20 °C. On the day of acquisition, ethanol was removed, and the cells were washed again and treated with 100 μL of 0.2 mg/mL Ribonuclease and followed by centrifugation at 2000 rpm for 5 min. The cells were resuspended in 200 μL 1X PBS and stained with 1 μL of 10 μg/mL PI and analyzed on a Guava EasyCyte 8 flow cytometer instrument.

### Chronic exposure

The cells were seeded in T25 flasks at a density of 4 × 10^5^ cells and incubated for 24 h. Subsequently, they were exposed to IC20 WSE and CSE for 24 h, followed by 24 h of recovery in regular media. We repeated this process twice a week, followed by passaging of the cells at the end of each week, for a total duration of 12 weeks. After 12 weeks of exposure, cells were collected in regular media and counted. These cells were considered as the heterogeneous populations in contrast to single-cell-derived colonies which were obtained through serial dilutions starting from the initial cell count to reach one cell per 100 µL. This cell suspension was seeded in a 96-well plate in at least 10 wells/condition to make sure that each colony is derived from one cell only. Plates were incubated and observed daily on the microscope until they divided and formed colonies. Colonies forming 50 cells or more were selected and transferred to bigger wells, then to flasks. We selected three colonies per condition. Cells were collected, frozen, or kept in culture for further experiments.

### RTL by qPCR

DNA was isolated from cells of heterogeneous populations and single-cell-derived colonies using the Flexigene kit (Qiagen, Germany) according to manufacturer’s protocol. The concentration and quality of isolated DNA samples were analyzed with a NanoDrop spectrophotometer (Denovix, Wilmington, United States). RTL was measured using quantitative PCR (qPCR) as per the Cawthon (2002) method on a CFX384 real time PCR instrument (BioRad, Germany). Telomere and *HBG* (hemoglobin subunit beta) primers were used (Supplementary Table S1). The PCR efficiency of each standard curve was estimated by the CFX manager software and was then used along with the threshold cycle (Ct) to calculate the T/S ratio according to the following equation:

$$\frac{{\log\left(Telomere\;efficiency\right)}^{Ct\;\left(Telomere\right)}}{{\log\left(SCG\;efficiency\right)}^{Ct\;\left(SCG\right)}}$$

### Human telomerase reverse transcriptase expression by qRT-PCR

RNA was isolated from heterogeneous cell populations using a Trizol-based protocol (Sigma-Aldrich, United States) followed by DNase treatment using the Invitrogen kit (Invitrogen, United States). The isolated RNA samples were analyzed with Nanodrop spectrophotometer (Denovix, Wilmington, United States) to measure concentration and purity. Additionally, we ran the RNA samples on a gel to assess their quality. Extracted RNA was converted to cDNA using the high capacity cDNA reverse transcription kit (Applied Biosystems, United States) according to the manufacturer’s protocol. cDNA was then used as a template for PCR amplification.

Telomerase expression was measured by quantitative reverse transcriptase PCR (qRT-PCR) with telomerase and *GAPDH* (glyceraldehyde-3-phosphate dehydrogenase) primers **(** Supplementary Table S1 **)** on a CFX384 real time PCR instrument (BioRad, Germany). Sensifast SYBR green master mix (Bioline, United States) was used. Six standards with concentrations ranging from 62.5 ng/μL to 0.02 ng/μL were prepared from a pool of cDNA samples using serial dilutions of 1:5, and were included on each plate along with a no-template control (NTC) which was RNAse-free water. Both samples and standards were run in triplicate, then relative expression with respect to the control was calculated using the ΔΔCt method.

### Telomerase activity by telomeric repeat amplification protocol (TRAP)

Proteins were extracted from heterogeneous cell populations with the lysis buffer (3 [(cholamidopropyl)-dimethyl-ammonium]-1-propanesulfonate (0.5% CHAPS) (Abcam, United States), 10 mM Tris-HCl (pH 7.5) and 5 mM β-mercaptoethanol (Bio-Rad, Germany),1 mM MgCl2, 1 mM EGTA, 0.1 mM [4 (2-aminoethyl)-benzenesulfonyl fluoride] hydrochloride and 10% glycerol (Sigma-Aldrich, United States) containing freshly added RNase inhibitor (Qiagen, Germany). A549 cells were incubated for 30 min on ice and vigorously vortexed every 10 min. After 30 min centrifugation at 4 °C and 14000 rpm, the supernatant containing the proteins was collected and the proteins were quantified with the Quick Start Bradford Protein Assay (BioRad, Germany).

We measured telomerase activity by real-time quantitative telomeric repeat amplification protocol (RTQ-TRAP) method which quantitatively measures telomerase activity in proteins using qPCR to detect telomerase-mediated extension of telomeric repeats (Wege et al., 2003). Forward TS and reverse ACX primers were used **(** Supplementary Table S1 **)**. Proteins extracted from HeLa cells, a positive control, were serially diluted to generate a standard curve. Heat inactivated samples were obtained by incubating some protein samples at 95 °C for 10 min prior to activity measurement and were used as No Activity Controls (NAC).

### Expression of EMT markers by qRT-PCR

The mRNA expression of some EMT markers was evaluated after 12 weeks of exposure in both heterogeneous populations and single-cell-derived colonies to assess the potential of cancer progression. The gene expression of the epithelial marker *CDH1* (Cadherin-1), in addition to the mesenchymal markers *SNAI1* (Snail Family Transcriptional Repressor 1), *SNAI2* (Snail Family Transcriptional Repressor 2), and *VIM* (Vimentin) were measured by qRT-PCR. The qRT-PCR mix included the sensifast SYBR Green master mix (Bioline, United States) and the specific forward and reverse primers for the epithelial or mesenchymal markers, with *GAPDH* as reference gene (Supplementary Table S1). Relative expression with respect to the control was calculated using the ΔΔCt method.

### Migration and invasion assays

The migration assay was done using two methods, the scratch wound-healing assay (Martinotti and Ranzato, 2020) and trans-well method. The wound-healing assay was done after 12 weeks of exposure in both heterogeneous populations and single-cell-derived colonies. We used 2% FBS media to inhibit cellular growth and proliferation. The cells seeded in a 6-well plate were scratched once they became confluent. Pictures of the wound were taken under the microscope at ×10 magnification at baseline and after 24 h. We measured the width of the wound with Perfect Screen Ruler windows application at 4 sites of the wound then calculated the average which we used to deduce the migrated distance.

Migration using trans-well inserts (Corning, United States) was done only on heterogeneous populations after 12 weeks of exposure. Cells were kept in FBS free DMEM at a concentration 1.4 × 10^5^ cell/mL. A total volume of 250 μL of cell suspension was added to the upper chamber, and 500 μL of DMEM with 10% FBS were added to the lower chamber. Following 24 h of incubation at 37 °C, media was discarded, and the cells were fixed with ice-cold methanol for 20 min. Migrating cells on the bottom side of the inserts were stained with Hoechst Dye (Thermo Fisher Scientific, United States). Prolong Antifade (Thermo Fisher Scientific, United States) was added to preserve the signal. Images were taken with Microscope Zeiss (Axio, Germany) at ×20 magnification. The cells were then counted using the ImageJ FIJI software (United States).

Invasion assay using trans-well inserts was also done on heterogeneous populations after 12 weeks of exposure. Matrigel (Corning, United States) was prepared on ice by diluting it as 1:5 ratio with ice-cold FBS-free DMEM, and 100 μL were spread onto the insert for coating. The plate was then incubated for 1 h at 37 °C. The following steps including cell seeding, incubation, fixation, staining, imaging, and counting were conducted the same way as for the migration assay. The percentage of migrating and invading cells was calculated based on the obtained count and the initial number of cells seeded. The investigators were blinded during the migration and invasion analyses to minimize measurement and counting bias.

### Statistical analysis

We had at least three independent biological replicates for each experiment (n ≥ 3). The resulting data are expressed as means +standard error of the mean (SEM). Individual data points are represented on all graphs with some experiments having six independent replicates. All analyses and figures were done on GraphPad Prism. Statistical analyses were conducted using non-parametric one-way ANOVA followed by Dunnett’s multiple comparisons test (Kruskal–Wallis) for RTL, telomerase, cell viability, apoptosis, sub-G0 and cell cycle. Statistical analysis for EMT, migration and invasion at week 12 were done using Mann-Whitney non-parametric two-tailed t-test. A P value of <0.05 indicated statistical significance. All significant P values are reported on the graphs.

## Results

### Sub-toxic acute exposure decreases cell viability without inducing cell death or cell cycle arrest in A549 cells

**FIGURE 1:**
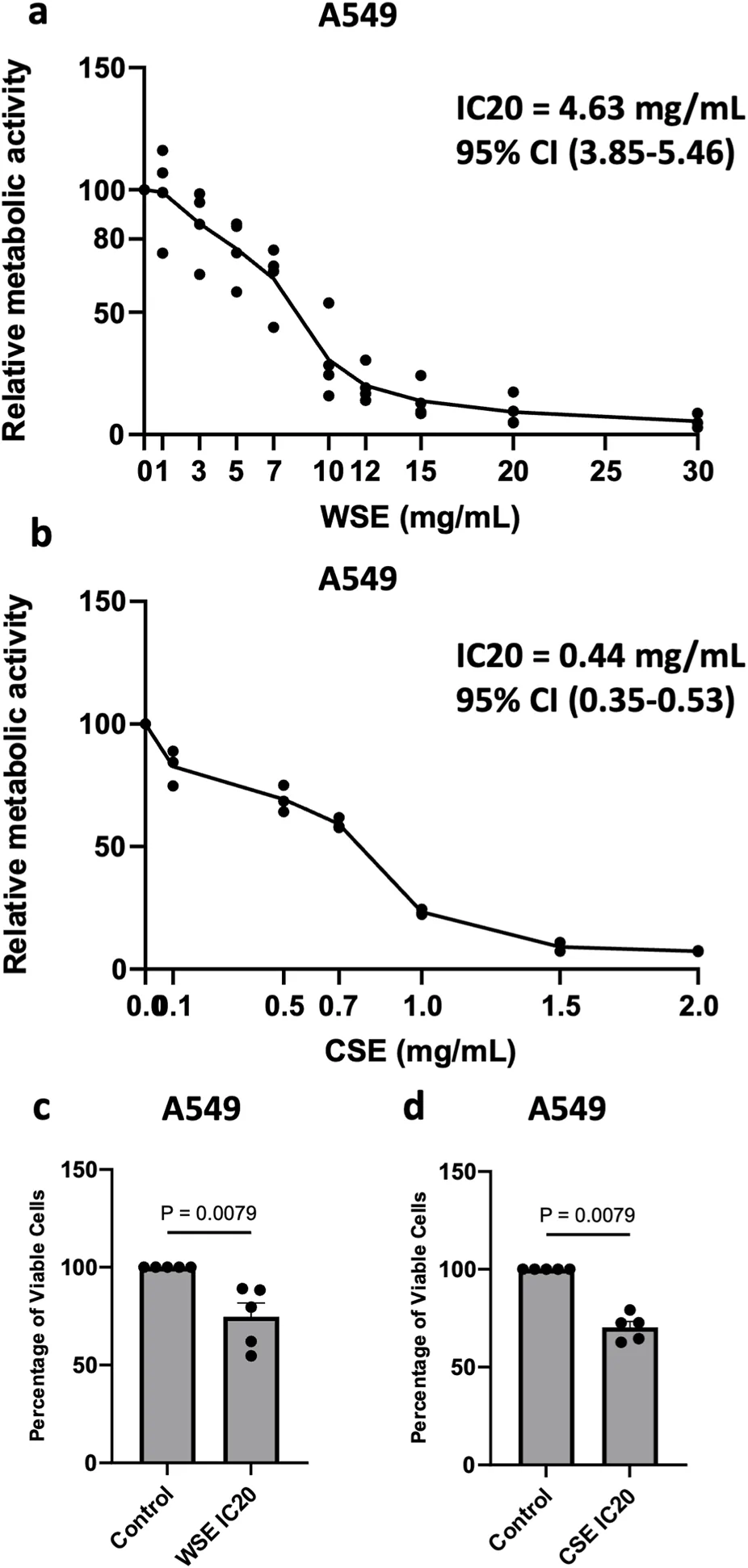
Determination of sub-toxic concentrations of WSE and CSE. The effect of 24 h of exposure to different concentrations of WSE and CSE on A549 cell metabolic activity (a,b) using MTT assay and the percentages of viable cells (c,d) after 24 h of exposure to IC20 concentrations of WSE and CSE with respect to the control using trypan blue assay. Data are means ± standard error (SEM) of at least three independent biological replicates (n # 3). Individual data points and statistically significant P values (P < 0.05) are reported on the graphs. Statistical analysis was done using Mann-Whitney non-parametric two-tailed t-test. IC20 and IC50 are the concentrations that respectively induce 20% and 50% decrease in cell metabolic activity.

The MTT results in Figure 1 showed that there was a dose-dependent decrease in the cells’ metabolic activity. The concentrations of WSE, (4.63 mg/mL; 95% CI (3.85–4.46)), and CSE (0.44 mg/mL; 95% CI (0.35–0.53)), which caused 20% cytotoxicity in the cells compared to the control at 24 h were determined as the IC20 concentrations (Figures 1a,b **)**. After 24 h of exposure, trypan blue results showed a significant decrease in cell viability which was significant but remained within the 20% expected range (P = 0.0079) for both WSE and CSE **(** Figures 1c,d). Trypan blue supplementary data at 24 h with IC50 concentrations (Supplementary Figure S1 **)** showed a more significant decrease in cell viability with IC50 WSE (7.75 mg/mL; 95% CI (6.97–8.55); P = 0.0050) and IC50 CSE (0.72 mg/mL; 95% CI (0.63–0.80); P = 0.0019), concentrations which caused 50% cytotoxicity compared to the control, but also within the 50% expected range **(** Supplementary Figures S1a, b **)**. The apoptosis assay done after 24 h of exposure showed no significant change with the IC20 concentrations, while it showed significant increase with IC50 CSE compared to the control (P = 0.0285) **(** Supplementary Figures S1c, d **)**. Additionally, cells in sub G0 showed no significant change in their percentage with IC20 concentrations while a significant increase was seen with the IC50 concentrations of WSE (P = 0.0225) and CSE (P = 0.0341) (Supplementary Figures S1e, f). However, the cell cycle phases were not affected, and no cell cycle arrest was seen after 24 h of exposure with any of the concentrations (Supplementary Figures S1g, h). **(** Supplementary Figures S1i–k **)** are representative figures for apoptosis, Sub G0 and cell cycle distributions respectively. These results show that the IC20 concentrations induced a significant decrease in cell viability that was within the 20% expected range and it is most likely due to apoptosis which was significant with the IC50 concentrations as shown by apoptosis and Sub G0 analysis. Thus, we considered IC20 as sub-toxic concentration and used it for the rest of the experiments. No morphological changes were seen after chronic exposure to WSE and CSE in any of the three cell-lines **(** Supplementary Table S2 **)**.

### Chronic exposure induces telomere shortening and telomerase activation in A549 heterogeneous/parents populations

**FIGURE 2:**
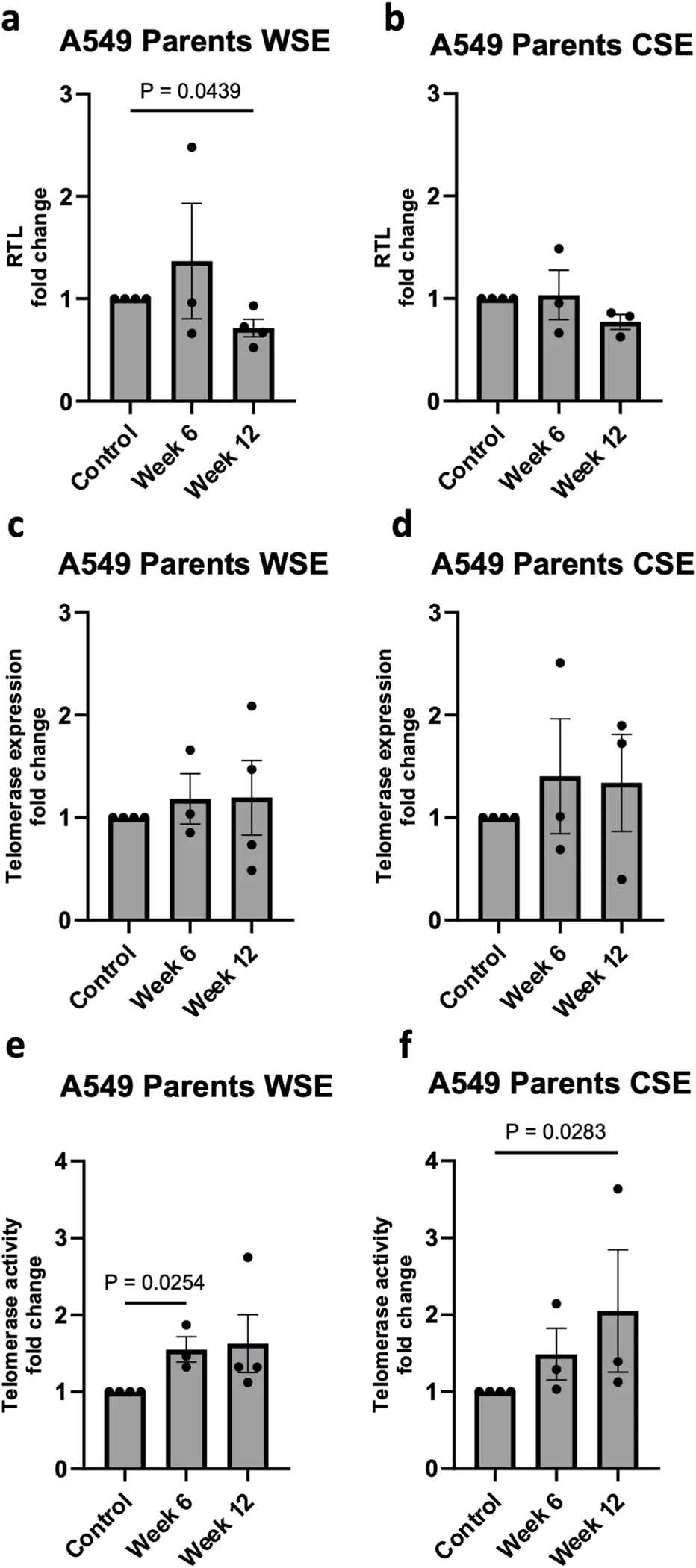
RTL, and telomerase expression and activity in heterogeneous/parents populations of A549. RTL (a,b), telomerase expression (c,d), and telomerase activity (e,f) of A549 heterogeneous/parents populations after 6 and 12 weeks of chronic exposure to IC20 concentrations of WSE and CSE with respect to the control. Data are means +standard error of the mean (SEM) of at least three independent biological replicates (n # 3). Individual data points and statistically significant P values (P < 0.05) are reported on the graphs. Statistical analysis was done using non-parametric one-way ANOVA followed by Dunnett's multiple comparisons test (Kruskal–Wallis). IC20 is the concentration that causes 20% decrease in cell metabolic activity.

To explore whether the length of the telomeres can be altered by the chronic exposure of WSE and CSE, we measured RTL and telomerase expression and activity after 6 and 12 weeks of exposure in the A549 heterogeneous/parents populations. Significant telomere shortening was seen after 12 weeks of exposure to waterpipe extract (P = 0.0439) but no significant change in RTL was observed after 6 and 12 weeks of chronic exposure to cigarette extract despite a trend of decrease (Figures 2a,b). No significant changes were seen in the mRNA expression of telomerase (Figures 2c,d). However, this was associated with a significant increase in telomerase activity at 6 weeks with WSE (P = 0.0254) and at week 12 with CSE (P = 0.0283) (Figures 2e,f). In single-cell-derived colonies, no significant change in telomere length was observed despite a trend of decrease with CSE **(** Supplementary Figure S2 **)**.

### Chronic exposure induces an increase in SNAI2 mesenchymal marker in both heterogeneous/parents populations and single-cell-derived colonies of A549 cells

**FIGURE 3:**
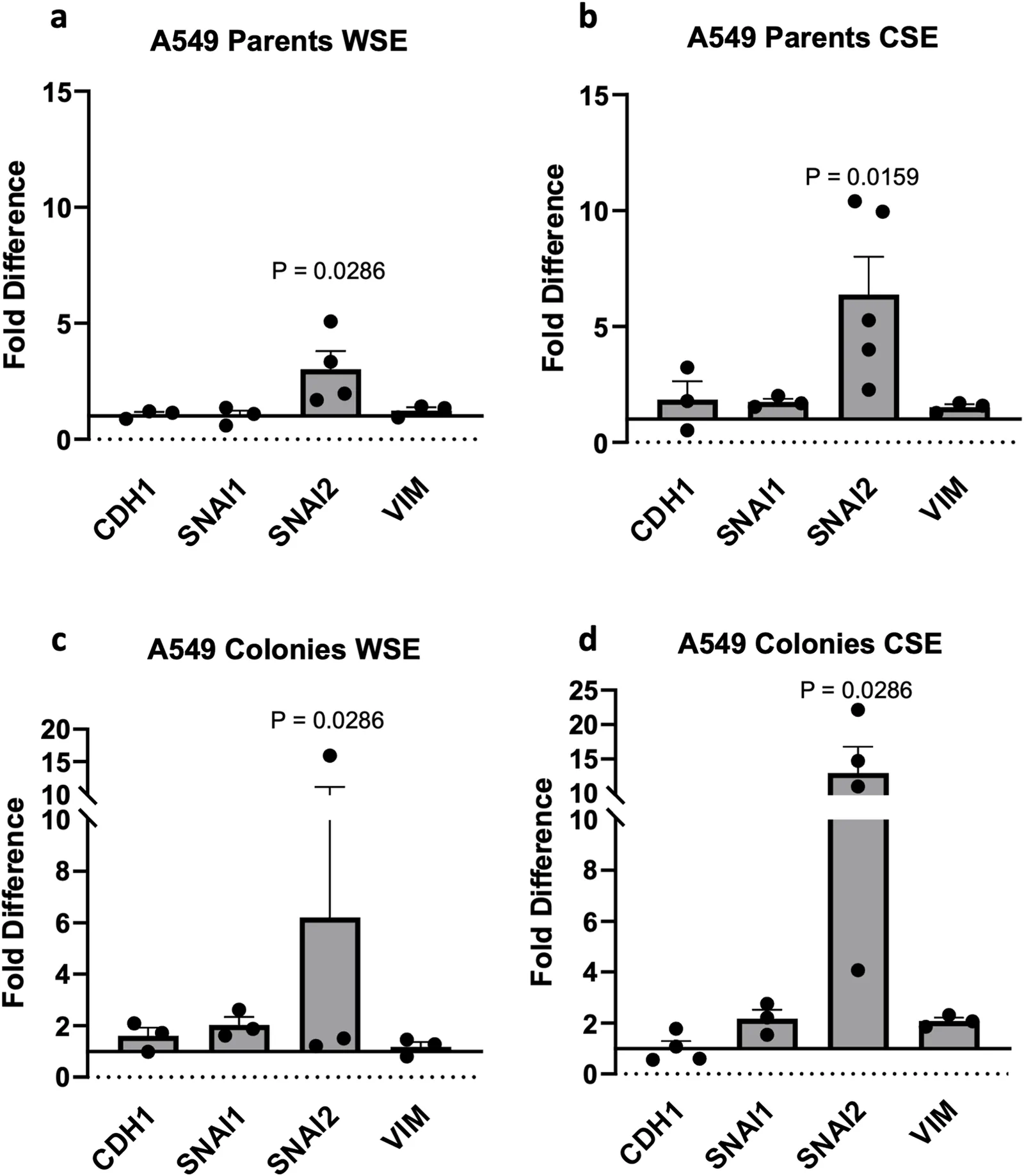
EMT in heterogeneous/parents populations and single-cell-derived colonies of A549. mRNA expression of EMT markers in the heterogeneous/parents populations exposed to WSE (a) and CSE (b), and in colonies exposed to WSE (c) and CSE (d) of A549 cells after 12 weeks of exposure to IC20 concentrations with respect to the control. Data are means +standard error of the mean (SEM) of at least three independent biological replicates (n # 3). Individual data points and statistically significant P values (P < 0.05) are reported on the graphs. Statistical analysis was done using Mann-Whitney non-parametric two-tailed t-test. IC20 is the concentration that causes 20% decrease in cell metabolic activity.

In heterogeneous/parents populations, 12 weeks of chronic exposure to both WSE and CSE resulted in a significant increase in *SNAI2* mRNA expression (P = 0.0286; P = 0.0159) (Figures 3a,b). Similarly, in single-cell-derived colonies, there was a significant increase, and to higher levels than in heterogeneous populations, in *SNAI2* with both WSE (P = 0.0286) and CSE (P = 0.0286) (Figures 3c,d).

#### Chronic exposure to CSE increases migration in single-cell-derived colonies of A549 cells

**FIGURE 4:**
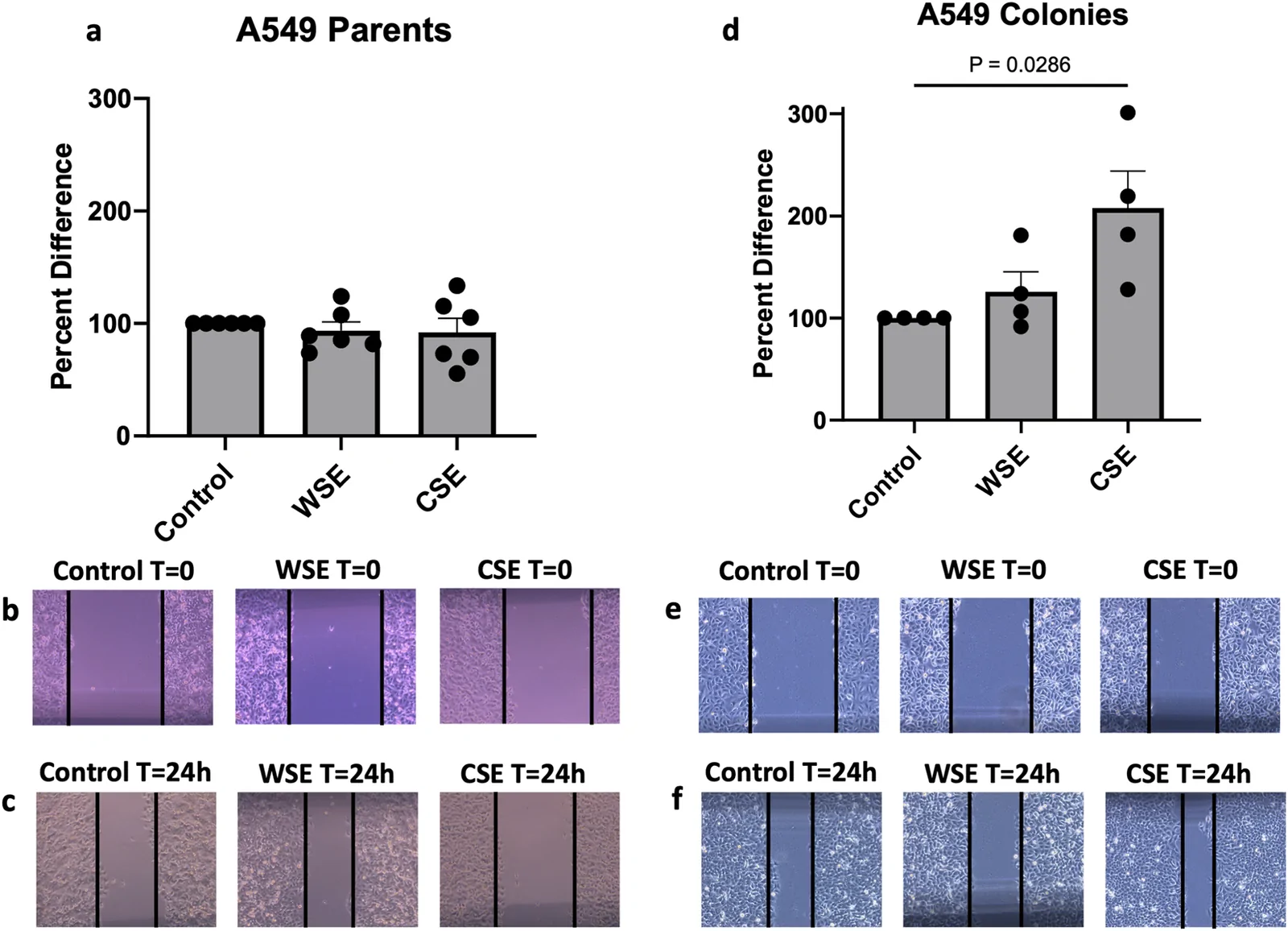
Migration assay in heterogeneous/parents populations and single-cell-derived colonies of A549. Migration percent difference in heterogeneous/parents populations **(a)** and colonies **(d)** of A549 and the corresponding representative figures of the wound healing at baseline **(b,e)** and after 24 h **(c,f)** after 12 weeks of chronic exposure to IC20 concentrations of WSE and CSE with respect to the control. Data are means +standard error of the mean (SEM) of at least three independent biological replicates (n ≥ 3). Individual data points and statistically significant P values (P < 0.05) are reported on the graphs. Statistical analysis was done using Mann-Whitney non-parametric two-tailed t-test. IC20 is the concentration that causes 20% decrease in cell metabolic activity.

The migration abilities of the heterogeneous populations, assessed by the wound-healing method, did not significantly change after 12 weeks of exposure to WSE and CSE (Figures 4a–c). Similarly, the migration and invasion abilities of the heterogeneous populations assessed by the trans-well assays did not show significant changes **(** Supplementary Figure S3 **)**. However, wound-healing assay on single-cell-derived colonies showed that CSE exposure resulted in significantly increased (P = 0.0286) migration (Figures 4d–f), but no significant changes were observed with WSE exposure in those cells (Figures 4d–f).

#### Results in validation cell-lines: breast and colon cancer cell-lines

In MCF7 colonies, there was no significant change in RTL after 6 and 12 weeks of chronic exposure to the IC20 concentrations of both WSE and CSE **(** Supplementary Figures S4a, b **)**. Significant increases in the mRNA expression of the mesenchymal marker *VIM* were seen with both exposures at 12 weeks (P = 0.0286) **(** Supplementary Figures S4c, d **)**. A non-significant trend of increase was observed in the migratory abilities of these cells assessed by the scratch assay **(** Supplementary Figures S4e–g **)**. As for HCT116 colonies, there was a significant decrease in RTL only at 6 weeks with chronic WSE exposure (P = 0.0458) **(** Supplementary Figures S5a, b **)**. Changes in mRNA expression of EMT markers at 12 weeks were not significant despite an increase in *VIM* **(** Supplementary Figures S5c, d **)**. However, HCT116 colonies showed a significantly decreased migratory ability with WSE exposure (P = 0.0286) **(** Supplementary Figures S5e, f **)** and a significant increase with CSE exposure (P = 0.0286) **(** Supplementary Figures S5e, g **).** MTT curves for MCF7 and HCT116 are reported with their specific IC20 concentrations and trypan blue cell viability results in Supplementary Figure S6.

## Discussion

This study represents the first investigation on the effects of chronic exposure to cigarette and waterpipe smoke extracts on relative telomere length, telomerase expression/activity and their potential association with cancer progression in a lung cancer cell line, with additional validations in colon and breast cancer cells. Additionally, we have demonstrated in this study an experimental method for tobacco smoke toxicity research that involves trapping machine-generated smoke on filters based on real-world human puff topography, preparing smoke extracts, and chronically exposing cells in culture to these extracts. While we focused on cigarette and waterpipe smoking, this model can be used to investigate an increasing number of other tobacco and nicotine smoking forms. Moreover, our study has translational value because by chronically exposing the cells to IC20 concentrations for up to 12 weeks, it would allow us to see chronic changes without immediate toxic effects, which reflects what happens in real-world chronic smoking and the effects it has on the long term.

While cigarettes and waterpipe have distinct composition and usage patterns (Jacob et al., 2013; Primack et al., 2018), our findings revealed that they have induced some similar cellular responses. However, it is worth noting that the IC20 concentration of waterpipe is 10 times higher than the one for cigarettes, highlighting a difference in potency between the two extracts. On the other hand, over a typical 1-h use bout, a waterpipe generates more TPM than a pack of cigarettes (Shihadeh, 2003), suggesting that the potency is not directly comparable. However, for this study, we normalized the concentrations of WSE and CSE to induce similar cytotoxic effects for each cell line whereby we used the concentrations that induced 20% and 50% cytotoxicity. This is consistent with other *in vitro* smoke-exposure studies on different cell lines that reported the use of non-cytotoxic/sublethal/sub-toxic concentrations of cigarette extract (Gugatschka et al., 2019; Reis et al., 2021; Stabile et al., 2018) or waterpipe condensate (Rammah et al., 2012; Zaarour et al., 2022) to maintain cell viability at around 70%–80% while still inducing measurable changes for a long-term analysis.

In this study, our focus is on lung cancer. We, however, added breast and colon cancer cells to evaluate the effect of CSE and WSE on other cancer models. We also used two types of cell populations, heterogeneous populations and single-cell-derived colonies. While the heterogeneous population reflects the natural diversity of the cultured cells and helps in measuring the average response, colonies allow to identify cell-specific behaviors (Lawson et al., 2018; Zhou et al., 2023).

Our results showed that chronic exposure altered the telomere and telomerase dynamics in A549 cells. WSE exposure induced significant telomere shortening at 12 weeks and significant telomerase activity increase at 6 weeks, while CSE exposure induced a trend of decrease in telomere length and a significant increase in telomerase activity at 12 weeks (Figure 2). A non-significant trend of decrease in telomere length was seen in single-cell-derived colonies, especially with CSE exposure **(** Supplementary Figure S2 **)**. These results suggest that smoke exposure can affect both telomeres and telomerase but in opposite directions; telomeres shorten upon exposure, while telomerase activity increases as a compensatory mechanism to prevent further shortening. While this hypothesis is biologically possible because telomerase recruitment depends on telomere length, and cancer cells tend to reactivate telomerase to stabilize telomere length and bypass senescence/apoptosis (Roake and Artandi, 2020; Robinson and Schiemann, 2022); it needs to be validated in additional cell lines, and a limitation of this study is that we did not measure telomerase activity in the MCF7 and HCT116 colonies.

Although our cell model lacks a tumor microenvironment, immune components, and systemic metabolism of smoke toxicants which limit its translational potential, our telomerase results in A549 align with three human subject studies, which have reported that smokers had increased telomerase activity in PBMCs (Marcon et al., 2017; Fernandes et al., 2021) and serum (Bozkus et al., 2017). This again suggests that telomerase can get activated as a compensatory mechanism to telomere attrition upon smoke exposure. Moreover, telomerase has telomere-independent functions, which include facilitating DNA repair, increasing apoptosis resistance, remodeling chromatin, and regulating gene expression, overall enhancing cell survival and stress resistance (Saretzki, 2009; Cong and Shay, 2008). This implies that telomerase could play an important role in promoting cancer progression. Moreover, we noticed that telomerase activity in the A549 cell-line significantly increased after chronic smoke exposure to both WSE and CSE, without significant changes at the mRNA level. This can be due to the many post-transcriptional mechanisms that regulate telomerase activity. In fact, hTERT (human telomerase reverse transcriptase) which is the core catalytic unit of telomerase can be subject to activating phosphorylation (Chang et al., 2006), nuclear translocation, protein stability versus degradation (Yang et al., 2023), and alternative splicing (Plyasova and Zhdanov, 2021). Also, telomerase holoenzyme assembly and recruitment to telomeres are necessary for its proper activity (Shepelev et al., 2023).

RTL and telomerase changes in A549 were associated, after 12 weeks of exposure, with significantly increased mRNA expression of the mesenchymal marker *SNAI2* in both heterogeneous populations and single-cell-derived colonies (Figure 3). Despite not seeing any significant changes in the migration abilities of A549 heterogeneous populations, CSE exposure has led to significantly higher migration in the colonies, while no significant change was observed with WSE (Figure 4). Comparable results were seen in the MCF7 and HCT116 colonies where the mRNA expression of the mesenchymal marker *VIM* showed a significant increase in MCF7 colonies **(** Supplementary Figure S4 **)** and a non-significant trend of increase in the HCT116 colonies **(** Supplementary Figure S5 **)** with both CSE and WSE. Also, migration in MCF7 colonies showed a trend of increase with both exposures **(** Supplementary Figure S4 **)** while CSE significantly increased the migration of HCT116 colonies. On the other hand, HCT116 exposed to WSE showed an unexpected decrease in migration requiring additional investigation which is out of the scope of this study **(** Supplementary Figure S5 **)**. However, it is worth noting that despite changes in migration, we did not notice any changes in the morphology of the cells **(** Supplementary Table S2 **)**. This is supported by the literature that migration increase can occur without obvious changes in morphology but rather through changes in cytoskeleton, polarity and focal adhesions (Merino-Casallo et al., 2022; Thapa et al., 2023).

We observed that chronic exposure for 12 weeks induced a significant increase in the mRNA expression of the mesenchymal markers *SNAI2* in A549 heterogeneous populations and colonies, and *VIM* in MCF7 colonies*.* While both *SNAI2* and *VIM* are mesenchymal markers and contribute to cancer progression through various mechanisms, their increase at the mRNA level without significant alterations in the rest of the markers does not indicate epithelial-to-mesenchymal transition. However, these results provide valuable insights that should be confirmed at the protein level especially that EMT markers are known to be extensively regulated post-transcriptionally and post-translationally (Kang et al., 2021; Migault et al., 2022).

The absence of significant changes in the migration of heterogeneous populations of A549 can be attributed to the heterogeneity of the various cell subpopulations which could have a variety of migratory abilities. As a result, an enhanced migration of a specific cell subpopulation can be diluted by the average response of the heterogeneous population (Lawson et al., 2018; Zhou et al., 2023). Therefore, by looking at single-cell-derived colonies, we can isolate the colonies that acquired enhanced migration due to the chronic smoke exposure, and it shows a more evident association. However, colonies can also reflect cell plasticity meaning that they may acquire certain phenotypic changes during the process of clonal expansion (Mathis et al., 2017), but in our case, we observed significantly enhanced migration with CSE exposure compared to the control in A549 and HCT116 colonies. This could indicate that chronic exposure might be enhancing, at a cell-by-cell level, migration or even plasticity, referred to as cancer stemness in some studies (Qian et al., 2018; Sharma et al., 2023).

Moreover, the fact that WSE did not affect the migration abilities in a similar way to CSE can be attributed first to their different chemical compositions according to the literature (Badran and Laher, 2020; Primack et al., 2016; Daher et al., 1994; Sepetdjian et al., 2010; Shihadeh et al., 2015), and second to the multifaceted nature of the EMT program. In fact, EMT is a highly complex process regulated by multiple pathways that influence cellular plasticity, invasion, and inflammation, often requiring intricate crosstalk between signaling cascades to drive functional outcomes (Pearson, 2019; Youssef et al., 2024). Additionally, the tumor microenvironment, including extracellular matrix components, stromal interactions, and molecular signals like growth factors (e.g., TGF-β, EGF), hormones, cytokines (e.g., IL-6, TNF-α), and chemokines plays a fundamental role in stimulating EMT (Pearson, 2019; Youssef et al., 2024); these factors are notably absent within our experimental conditions, limiting the translation of EMT markers expression into functional changes in cellular behavior.

Both cigarette and waterpipe smoke are known to contain PAHs and heavy metals and the chronic exposure to PAHs (Li et al., 2025; Pavanello et al., 2010), cadmium (Wai et al., 2024), and other heavy metals (Chen et al., 2024) has been linked to telomere shortening in peripheral blood mononuclear cells (PBMCs) in humans, newborns and rats, accompanied with significant oxidative stress, inflammation, genetic, epigenetic, and chromosomal changes. In accordance with our RTL data, two other studies on different cell-lines, including human non-small cell lung carcinoma, human primary lung fibroblasts, and mouse embryonic stem cells, also reported significant telomere shortening after 2 weeks of exposure to low doses of CSE or cadmium (Deb et al., 2024; Huang et al., 2013). Additionally, two animal model studies showed similar results of telomere shortening in early mouse embryos *in vitro* exposed to CSC (Huang et al., 2010) and blastocysts of female CD1 mice exposed to direct cigarette smoke or CSC by insufflation (Huang et al., 2009). Moreover, several human subject studies have also reported that smoking is strongly associated with accelerated telomere shortening in many types of human cells, including leukocytes, lymphocytes, and small airway epithelial cells (Walters et al., 2014; Astuti et al., 2017; Wulaningsih et al., 2016; Muezzinler et al., 2015). Collectively, these studies show triangulated evidence from multiple systems, including human studies (epidemiology) and experimental cellular and animal models. To the best of our knowledge, there are no published articles investigating the effect of smoke exposure on telomeres/telomerase in breast and colon cells. However, there are studies that showed that cigarette or waterpipe smoke exposure induces EMT and increases migration, invasion, and metastatic abilities of breast and colon cancer cells (Di Cello et al., 2013; Kim et al., 2017; Sadek et al., 2018).

A limitation of this study is the focus on one lung cell line and a cancerous one while it would be beneficial in the future to include additional normal and cancer lung cell lines such as lung squamous cell carcinoma cells for example, which represent a type of lung cancer that is heavily associated with smoking (Lee et al., 2012). This would allow for more genetic diversity and comparisons across cell lines. While we focused in this study mainly on cancer progression, studying normal cell lines would allow us to look better at the early stages of cancer initiation and development through the telomere/telomerase and EMT dynamics. Additionally, the EMT markers were only assessed at the mRNA level; while they provide valuable insights, they should be confirmed at a protein level in future studies to strengthen the findings. Furthermore, telomerase expression/activity should be assessed in the single-cell-derived colonies of the three cancer cell lines. Moreover, telomere and telomerase mechanistic studies such as measuring DNA damage, oxidative stress, genomic instability and inhibiting telomerase, would be interesting to tackle in the future to examine whether telomere shortening and telomerase activation are actual drivers of cancer progression.

## Conclusion

Collectively, we investigated the effects of chronic exposure to WSE and CSE on telomeres, telomerase and cancer progression in the A549 lung cancer cell line with additional validation in two other cell lines. The 12 weeks chronic exposure in A549 heterogeneous populations resulted in significant telomere shortening with WSE and increased telomerase activity with CSE. Moreover, we found that chronic exposure to WSE and CSE induced significant increase in the mRNA expression of *SNAI2* mesenchymal marker in heterogeneous populations, and to higher levels in single-cell-derived colonies of A549 cells, which are to be validated at the protein level. A significant increase in migration was also observed with CSE exposure in the colonies. Further assessment of RTL, EMT and migration was done in MCF7 breast cancer and HCT116 colon cancer cells. MCF7 colonies showed no change in RTL, but a significant increase in the mesenchymal marker *VIM*, with no change in migration, while HCT116 colonies showed significant RTL shortening at 6 weeks with WSE, a significant decrease in migration with WSE and a significant increase in migration with CSE, but no change in EMT markers. Further work is needed to integrate additional normal and lung cancer cell lines, more mechanistic telomeres/telomerase experiments, assess telomerase in single-cell-derived colonies of the three cell lines, and confirm EMT at the protein level.

## Summary

This article focuses on the effect of chronic exposure to cigarette and waterpipe smoke extracts on telomere length, telomerase expression, telomerase activity, EMT markers and migration abilities in lung cancer cells with some validations in breast, and colon cancer cell lines.

## Data Availability

The original contributions presented in the study are included in the article/Supplementary Material, further inquiries can be directed to the corresponding authors.

## Supplementary material

SUPPLEMENTARY FIGURE S1 Trypan blue, apoptosis, and cell cycle assays in A549 cells. Percentages of A549 viable cells **(a, b)** after 24 h of exposure to IC20 and IC50 concentrations of WSE and CSE with respect to the control using trypan blue assay. Percentage distributions of A549 live, early apoptotic, and late apoptotic cells **(c, d)** after 24 h of exposure to IC20 and IC50 concentrations of WSE and CSE. Percentages of A549 cells in Sub G0 phase **(e, f)** after 24 h of exposure to IC20 and IC50 concentrations of WSE and CSE. A549 cell cycle distributions after 24 h of exposure to IC20 and IC50 concentrations of WSE and CSE, using PI flow cytometry **(g, h)**. **(i, j, k)** are representative figures for apoptosis, Sub G0, and cell cycle distributions respectively, where red circles in **(j)** show dead cells. Data are means +standard error of the mean (SEM) of at least three independent biological replicates (n ≥ 3) except for 1.5 and 2 mg/mL CSE whereby there were only two replicates. Individual data points and statistically significant P values (P < 0.05) are reported on the graphs. Statistical analysis was done using non-parametric one-way ANOVA followed by Dunnett’s multiple comparisons test (Kruskal-Wallis). IC20 and IC50 are the concentrations that respectively induce 20% and 50% decrease in cell metabolic activity.

SUPPLEMENTARY FIGURE S2 RTL in A549 single-cell-derived colonies. RTL of A549 single-cell-derived colonies after 6 and 12 weeks of chronic exposure to IC20 concentrations of WSE **(a)** and CSE **(b)** with respect to the control. Data are means +standard error of the mean (SEM) of three independent biological replicates (n = 3). Individual data points and statistically significant P value (P < 0.05) are reported on the graphs. Statistical analysis was done using non-parametric one-way ANOVA followed by Dunnett’s multiple comparisons test (Kruskal-Wallis). IC20 is the concentration that causes 20% decrease in cell metabolic activity.

SUPPLEMENTARY FIGURE S3 Trans-well migration and invasion assays in A549 heterogeneous populations. Migration percentages **(a, b)**, and invasion percentages **(c, d)**, and migration **(e)** and invasion **(f)** representative figures of A549 heterogeneous populations after 12 weeks of chronic exposure to IC20 concentrations of WSE and CSE with respect to the control. Data are means +standard error of the mean (SEM) of at least three independent biological replicates (n ≥ 3). Individual data points are reported on the graphs. Statistical analysis was done using Mann-Whitney non-parametric two-tailed t-test. IC20 is the concentration that causes 20% decrease in cell metabolic activity.

SUPPLEMENTARY FIGURE S4 RTL, EMT, and migration in MCF7 single-cell-derived colonies. RTL fold change **(a, b)**, fold difference of mRNA expression of EMT markers **(c, d)**, migration percent difference **(e)** and representative figures of scratch assay **(f, g)** of MCF7 single-cell-derived colonies after 12 weeks of chronic exposure to IC20 concentrations of WSE and CSE with respect to the control. Data are means +standard error of the mean (SEM) of at least three independent biological replicates (n ≥ 3). Individual data points and statistically significant P values (P < 0.05) are reported on the graphs. Statistical analysis was done using non-parametric one-way ANOVA followed by Dunnett’s multiple comparisons test (Kruskal-Wallis) for RTL, and Mann-Whitney non-parametric two-tailed t-test for EMT and scratch assay. IC20 is the concentration that causes 20% decrease in cell metabolic activity.

SUPPLEMENTARY FIGURE S5 RTL, EMT, and migration in HCT116 single-cell-derived colonies. RTL fold change **(a, b)**, fold difference of mRNA expression of EMT markers **(c, d)**, migration percent difference **(e)** and representative figures of scratch assay **(f, g)** of HCT116 single-cell-derived colonies after 12 weeks of chronic exposure to IC20 concentrations of WSE and CSE with respect to the control. Data are means +standard error of the mean (SEM) of at least three independent biological replicates (n ≥ 3). Individual data points and statistically significant P values (P < 0.05) are reported on the graphs. Statistical analysis was done using non-parametric one-way ANOVA followed by Dunnett’s multiple comparisons test (Kruskal-Wallis) for RTL, and Mann-Whitney non-parametric two-tailed t-test for EMT and scratch assay. IC20 is the concentration that causes 20% decrease in cell metabolic activity.

SUPPLEMENTARY FIGURE S6 MCF7 and HCT116 MTT curves and trypan blue cell viability assays. The effect of 24 h of exposure to different concentrations of WSE and CSE on MCF7 **(a, b)** and HCT116 **(c, d)** cell metabolic activity using MTT assay. Percentages of MCF7 **(e, f)** and HCT116 **(g, h)** viable cells after 24 h of exposure to IC20 and IC50 concentrations of WSE and CSE with respect to the control using trypan blue assay. Data are means ± standard error (SEM) of at least three independent biological replicates (n ≥ 3) except for MTT of MCF7 1.5 and 2 mg/mL CSE whereby there were only two replicates. Individual data points and statistically significant P values (P < 0.05) are reported on the graphs. IC20 is the concentrations that induces 20% decrease in cell metabolic activity.

SUPPLEMENTARY TABLE S1 Primers used for molecular assays.

SUPPLEMENTARY TABLE S2 Morphology pictures. Representative pictures for morphology of A549, MCF7 and HCT116 cells at baseline and after chronic exposure (6 and 12 weeks) to IC20 waterpipe (WSE) and cigarette (CSE) smoke extract at 10X and 40X magnifications. IC20: 20% Inhibitory Concentration. (a) A549 10X; (b) A549 40X; (c) MCF7 10X; (d) MCF7 40X; (e) HCT116 10X; (f) HCT116 40X.
