# Supplementary material for: The impact of chronic exposure to cigarette and waterpipe smoke extracts on telomeres, telomerase, and cancer progression
Source: Front Genet. 2026 Sep 9;17:1837683. doi: 10.3389/fgene.2026.1837683 (PMC13597059; doi:10.3389/fgene.2026.1837683)
Supplement: Supplementary file 1 [file DataSheet1.pdf]

S1 Table. Primers used for molecular assays.

	Primer	Sequence	
		**Forward**	**Reverse**
**RTL**	Telomere	ACACTAAGGTTTGGGTTTGGGTTTGGGTTTGGGTTAGTGT	TGTTAGGTATCCCTATCCCTATCCCTATCCCTATCCCTAACA
	HBB	GCTTCTGACACAACTGTGTTCACTAGC	CACCAACTTCATCCACGTTCACC
**hTERT mRNA Expression**	*GAPDH*	GAAGGTGAAGGTCGGAGTC	GAAGATGGTGATGGGATTTC
	Telomerase	CGTCGAGCTGCTCAGGTCTT	AGTGCTGTCTGATTCCAATGCTT
**Telomerase Activity**	Forward TS	AATCCGTCGAGCAGAGTT	-
	Reverse ACX	-	GCGCGG (CTT ACC)3CTAACC
**EMT Markers**	*CDH1*	CAGAAAGTTTTCCACCAAAG	AAATGTGAGCAATTCTGCTT
	SNAI1	AAGATGCACATCCGAAGCCA	CTTCTCGCCAGTGTGGGTC
	SNAI2	GAACTCACACGGGGGAGAAG	ACACAGCAGCCAGATTCCTC
	VIM	AGGTGGACCAGCTAACCAAC	TCTCCTCCTGCAATTTCTCC

RTL: Relative Telomere Length

HBB: Hemoglobin Subunit Beta

hTERT: Human Telomerase Reverse Transcriptase

GAPDH: Glyceraldehyde-3-Phosphate Dehydrogenase

TS: Forward TS Primer

ACX: Reverse ACX Primer

EMT: Epithelial-to-Mesenchymal Transition

***CDH1*:** Cadherin-1

***SNAI1*:** Snail Family Transcriptional Repressor 1

***SNAI2*:** Snail Family Transcriptional Repressor 2

***VIM*:** Vimentin
