# Supplementary material for: The impact of chronic exposure to cigarette and waterpipe smoke extracts on telomeres, telomerase, and cancer progression
Source: Front Genet. 2026 Sep 9;17:1837683. doi: 10.3389/fgene.2026.1837683 (PMC13597059; doi:10.3389/fgene.2026.1837683)
Supplement: Supplementary file 2 [file DataSheet2.docx]

| **S2 Table a. Morphology pictures of A549 cells, 10X magnification.** | | | |
| --- | --- | --- | --- |
|  | **Baseline** | **Week 6** | **Week 12** |
| **Control** |  |  |  |
| **WSE IC20** |  |  |  |
| **CSE IC20** |  |  |  |

| **S2 Table b. Morphology pictures of A549 cells, 40X magnification.** | | | |
| --- | --- | --- | --- |
|  | **Baseline** | **Week 6** | **Week 12** |
| **Control** |  |  |  |
| **WSE IC20** |  |  |  |
| **CSE IC20** |  |  |  |

| **S2 Table c. Morphology pictures for MCF7 cells, 10X magnification.** | | | |
| --- | --- | --- | --- |
|  | **Baseline** | **Week 6** | **Week 12** |
| **Control** |  |  |  |
| **WSE IC20** |  |  |  |
| **CSE IC20** |  |  |  |

| **S2 Table d. Morphology pictures of MCF7 cells, 40X magnification.** | | | |
| --- | --- | --- | --- |
|  | **Baseline** | **Week 6** | **Week 12** |
| **Control** |  |  |  |
| **WSE IC20** |  |  |  |
| **CSE IC20** |  |  |  |

| **S2 Table e. Morphology pictures of HCT116 cells, 10X magnification.** | | | |
| --- | --- | --- | --- |
|  | **Baseline** | **Week 6** | **Week 12** |
| **Control** |  |  |  |
| **WSE IC20** |  |  |  |
| **CSE IC20** |  |  |  |

| **S2 Table f. Morphology pictures of HCT116 cells, 40X magnification.** | | | |
| --- | --- | --- | --- |
|  | **Baseline** | **Week 6** | **Week 12** |
| **Control** |  |  |  |
| **WSE IC20** |  |  |  |
| **CSE IC20** |  |  |  |
